# Cloud Inspired White and Grey Plasmonic Metasurfaces for Camouflaged Thermal Management

**DOI:** 10.1002/adma.202501080

**Published:** 2025-06-06

**Authors:** Mhd Adel Assad, Moheb Abdelaziz, Torge Hartig, Thomas Strunskus, Alexander Vahl, Franz Faupel, Mady Elbahri

**Affiliations:** ^1^ Nanochemistry and Nanoengineering School of Chemical Engineering Department of Chemistry and Materials Science Aalto University Espoo Finland; ^2^ Institute for Materials Science Chair for Multicomponent Materials Faculty of Engineering Kiel University Kiel Germany

**Keywords:** cloud, metasurfaces, mimicking, plasmonic, polarizonic, white

## Abstract

Inspired by nature's color‐driven thermal regulation mechanisms and the atmospheric radiative effects of cloud‐aerosol interactions, this work presents the design of disordered metasurfaces capable of achieving white and grey plasmonic colors. This innovation advances light and thermal management technologies within the framework of stealth and camouflage applications. The white plasmonic metasurfaces emulate the cooling effects of clouds, reducing substrate temperatures by a relative −10 °C under standard solar illumination through backscattering. In contrast, transitioning to a grey state with a nanocomposite absorber suppresses backscattering and enables efficient light trapping, resulting in a relative +10  °C temperature increase compared to conventional black absorbers. These findings introduce a novel approach to localized thermal management, distinct from traditional passive cooling strategies that rely on high‐emissivity materials. The metasurfaces’ low‐emissivity properties and visible appearance open opportunities in advanced camouflage, stealth technologies, and thermal energy solutions. Additionally, the scalable, sustainable design, realized through all‐in‐chamber nanofabrication via sputtering, eliminates the need for chemically intensive synthesis methods while ensuring long‐term stability.

## Graphical abstract

Inspired by clouds' role in Earth's thermal management, this work presents disordered plasmonic metasurfaces that mimic their radiative effects. The white metasurface enhances backscattering, acting as a cooling layer with camouflage properties. The grey state suppresses backscattering and enhances light trapping, surpassing conventional black absorbers. These metasurfaces enable advancements in camouflage, low‐emissivity coatings, and thermal infrared stealth technologies.

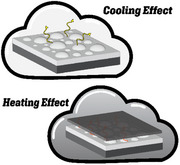

## 1 Introduction

In nature, white and grey colors in living organisms are critical for thermal regulation and camouflage, with many species possessing these traits to better adapt to their environments and remain concealed from predators.^[^ ^1^, ^2^, ^3^, ^4^, ^5^ ^]^ For instance, polar bears have white fur that allows them to blend into snowy landscapes and scatter sunlight, which reduces heat absorption and keeps them cool, particularly during the long days of the Arctic summer.^[^ ^6^, ^7^ ^]^ Similarly, grey‐colored animals, like certain birds and mammals, use their grey tones to blend into rocky or shaded areas, improving their camouflage.^[^ ^8^, ^9^ ^]^ These grey animals also have bodies that can absorb a wide range of the solar spectrum, which helps them manage heat by trapping and balancing it effectively.^[^ ^10^, ^11^, ^12^ ^]^

Analogously, and on a different length scale, this interplay of white and grey mechanisms extends beyond living organisms to the Earth's atmosphere, where it is governed by radiative forcing principles.^[^ ^13^, ^14^ ^]^ These principles highlight a model for advanced thermal management by quantifying changes to the balance of energy flowing through the atmosphere. In this context, clouds and aerosols are crucial in regulating Earth's energy balance.^[^ ^15^, ^16^ ^]^ White clouds act as natural cooling agents by backscattering sunlight back into space.^[^ ^17^ ^]^ Aerosols, on the other hand, have more complex interactions and can be categorized into two main types, namely scatterers and absorbers.^[^ ^18^ ^]^ Absorbing aerosols promote heating by trapping solar energy whereas the overall impact of aerosols depends on their type and their interactions with clouds. For instance, when absorbing aerosols are positioned above clouds, they can interfere with the cooling effect by counteracting backscattering. This results in an overall heating effect and contributes to the formation of a “grey cloud” phenomenon. It is important to note that the heat generated by light absorption from aerosols differs fundamentally from the greenhouse effect. Aerosols primarily interact with light in the visible and ultraviolet (UV) spectrum, while greenhouse gases mainly absorb infrared (IR) radiation. This distinction means that while greenhouse gases trap heat by absorbing IR radiation and contributing to warming, aerosols can either cool or heat the atmosphere depending on their properties and configuration. Higher backscattering by aerosols, which reflects more sunlight into space, supports the cooling of the climate system.^[^ ^19^, ^20^ ^]^

In the context of color functionality, plasmonic materials as well as metasurfaces have been extensively employed to manipulate light via scattering and absorption, enabling the creation of vibrant colors and black materials that are exploited in a variety of applications, including energy absorption,^[^ ^21^, ^22^, ^23^, ^24^, ^25^, ^26^, ^27^, ^28^ ^]^ anti‐counterfeiting,^[^ ^29^, ^30^, ^31^ ^]^ stealth technologies,^[^ ^32^, ^33^, ^34^ ^]^ and beyond.^[^ ^35^, ^36^, ^37^, ^38^, ^39^, ^40^, ^41^ ^]^ Despite these advances, achieving a grey and particularly white appearance using plasmonic materials remains a major challenge as plasmonic materials are naturally strong absorbers,^[^ ^42^, ^43^, ^44^, ^45^ ^]^ making them highly effective for applications such as broadband solar absorbers^[^ ^28^, ^41^, ^46^, ^47^, ^48^, ^49^ ^]^ that use metallic or dielectric metamaterials.^[^ ^50^, ^51^, ^52^, ^53^, ^54^, ^55^ ^]^ Cooling is typically associated with white materials, which are absent from the plasmonic color spectrum. The term “white materials” refers to substances that scatter light diffusely across all wavelengths, giving them a white appearance, as seen in titanium dioxide used in paint. This diffuse scattering is different from backscattering, which refers to reflections directed backward relative to the incident light. The whiteness of a material results from weak light localization and multiple scattering events within a random medium, leading to diffuse reflection.^[^ ^56^ ^]^ However, achieving backscattering is particularly difficult for both dielectric and plasmonic metals.^[^ ^57^, ^58^, ^59^, ^60^ ^]^ In the context of cooling, passive coolers also suffer from multiple drawbacks, for example, they can only provide passive cooling at night because of their unselectivity, which makes them ineffective under solar illumination.^[^ ^61^ ^]^ Due to these limitations, photonic radiative cooling strategies have been developed,^[^ ^62^ ^]^ but these often come at the expense of maintaining an authentic white color. A major disadvantage of high emissivity materials is their detectability in thermal IR, which makes thermal camouflage challenging. In addition, these materials are highly susceptible to ultraviolet exposure, which can cause degradation or unwanted optical excitations that reduce their long‐term stability and cooling efficiency. To achieve a white cooling surface while remaining undetectable in the thermal infrared range, a new optimized design is required that incorporates backscattering and a low emissivity coating therefore offering a novel and largely unexplored area. This endeavor explores harnessing color functionality with unique optical and thermal properties, paving the way for advanced material designs that balance scattering and absorption, with applications in substrate cooling, adaptive surfaces, energy‐efficient coatings, thermal regulation, and stealth technologies.

Drawing inspiration from the intricate interplay between clouds and aerosols in Earth's atmosphere, we have developed an artificial system designed to replicate these natural processes for precise control over the visual and thermal behavior with novel white and grey plasmonic design. Our design draws inspiration from the interplay of backscattering and its cancellation observed in cloud‐aerosol systems. Specifically, we mimic the white backscattering of clouds through a plasmonic metasurface and its transition to reduced backscattering (or greying) through the addition of a nanocomposite layer. While the transformation of clouds from white to grey due to aerosols has been treated as a simple and intuitive idea in the literature, our experiments provide the first concrete realization of this phenomenon at vastly different material properties and length scales. The white plasmonic metasurfaces resembling a cloud can effectively backscatter the solar radiation leading to a cooling effect that reduces the temperature of the underlying substrate by a relative −10 °C. Furthermore, by emulating the atmospheric layering effect, we incorporate a nanocomposite absorber on top of the white metasurface, which cancels the backscattering, resulting in a transition to a grey color and efficient light trapping. This enhancement leads to a significant temperature increase in the system, outperforming both conventional black and selective black absorbers by a relative +10 °C under 1 sun. Our pioneering design and fabrication of a plasmonic backscattering system, guided by both simulation and experimental measurements, bridges the gap between thermal efficiency and substrate thermal control, unlocking the transformative potential for advanced camouflage, stealth technologies, and innovative thermal energy solutions. This manuscript begins by introducing the design motivation, inspired by cloud and aerosol mechanisms associated with white and grey colors, followed by the demonstration of mimetic samples exhibiting these characteristics. We then present a detailed discussion of the technical aspects, simulations, and underlying mechanisms that explain the observed effects, concluding with the thermal characterization of the structures.

## 2 Results and Discussion

### 2.1 Design Principles Inspired by White and Grey Atmospheric Phenomena

**Figure 1:**
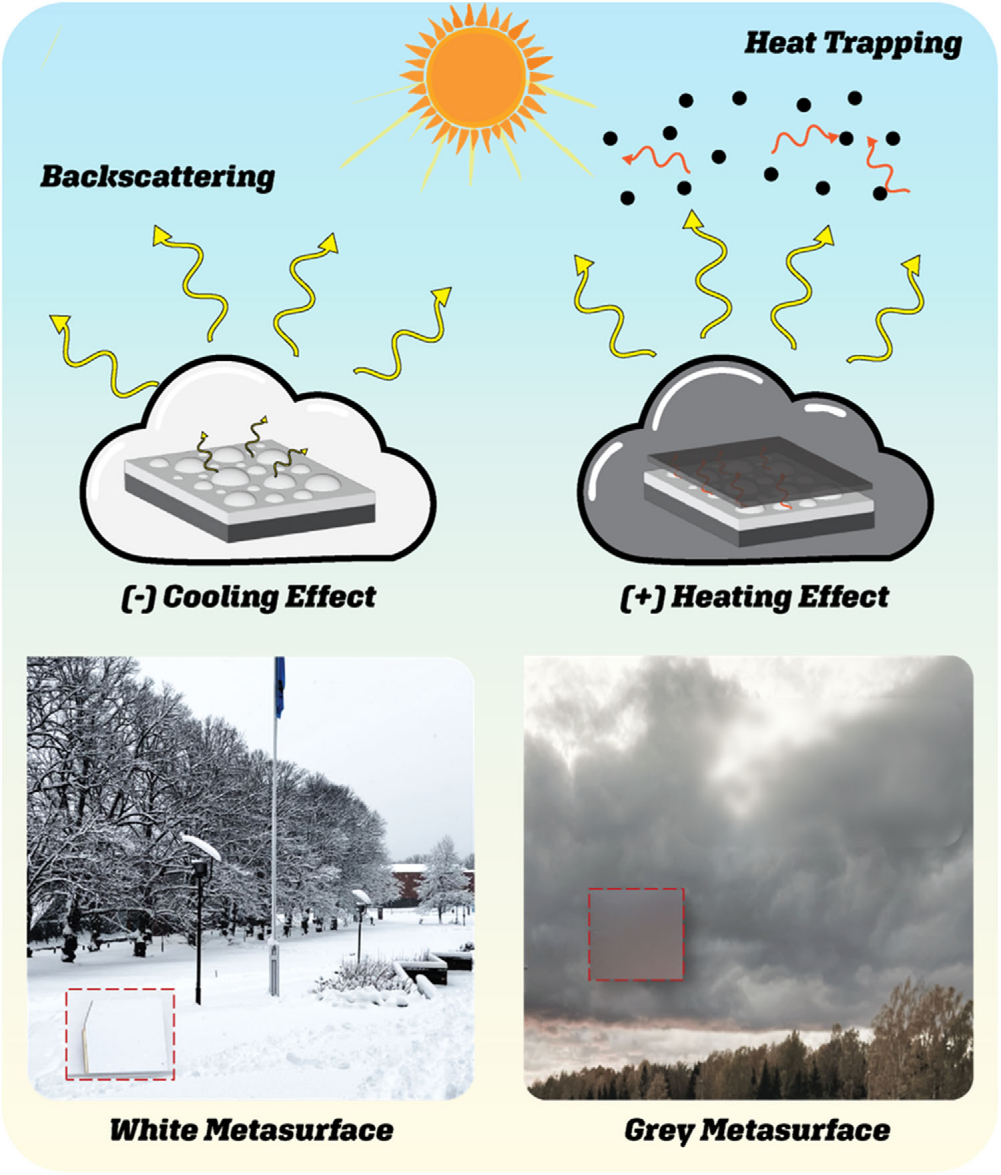
Design principle of the atmospheric‐inspired white and grey metasurfaces with camouflaged cloud‐mimicking thermal effects. The left panels show the white metasurfaces which mimics the white cloud by its backscattering and resulting in a cooling effect. In contrast, the right panel shows the grey metasurface counterpart mimicking the grey clouds and resulting in a heating effect due to heat trappment via clound‐aresol interaction. Both metasurfaces are visually overlaid onto natural scenes, snowy landscapes with white clouds and darker skies with grey clouds, thus demonstrating their effective camouflage in different atmospheric environments.

The concept of radiative forcing, which modulates the energy balance between absorbed and backscattered radiation, provides a foundational framework for understanding natural processes that regulate climate.^[^ ^63^ ^]^ This balance is essential for maximizing either cooling or heating efficiencies, depending on the net interactions of atmospheric constituents such as clouds and aerosols. These components, whether absorbing or scattering, influence the overall energy budget by contributing to absorption and backscattering processes. The interaction of these elements dictates how much solar energy is retained or reflected, significantly impacting the thermal behavior of the atmosphere. This complex interplay can be simplified into an equation that quantifies radiative forcing and its effects on climate regulation:

(1) $$Q_{\mathrm{net}}=Q_{\mathrm{abs}}-Q_{\mathrm{Bstr}}$$

where 𝑄_net_ represents the overall energy change. The terms 𝑄_abs_ and 𝑄_Bstr_, represent the energy contributions from absorbed and backscattered solar radiation, respectively. When Q_net_ is positive, it indicates that absorption is dominating over backscattering, resulting in a heating effect as more solar energy is trapped within the system. Conversely, when Q_net_ is negative, backscattering is the dominant process, leading to a cooling effect as more solar energy is reflected away from the system, reducing the net heat retained. This dynamic is illustrated in (**Figure** **1**), which shows a cooling response characterized by negative Q_net_ for a white cloud, and a positive heating response when an absorbing aerosol layer is present atop the cloud, canceling backscattering and inducing a heating effect. We realized a similar analogy through the structural design of the first white plasmonic silver. The backscattering and reflection across the visible to near‐infrared (NIR) spectrum are maximized using the metasurface, achieving a camouflage effect that integrates seamlessly with cloud and snowy environments (Figure 1). This choice of color is particularly suited for cooling applications, as white surfaces reflect a broad range of solar and thermal wavelengths, thereby limiting heat absorption and promoting temperature regulation as it will be shown below. This contrasts with highly emissive white paints, which, despite their scattering properties, tend to retain heat inefficiently and degrade under UV light. A distinctive feature of our design is the simulation of grey coloration akin to that observed in natural cloud‐aerosol interactions, a phenomenon not extensively studied. This effect is replicated using white metamaterials combined with a nanocomposite layer, emulating the complex interactions seen in cloud‐aerosol systems. The result is a grey appearance (Figure 1) created through a controlled combination. of broad‐spectrum light absorption and by the white scatterer. This grey transition represents a balanced approach, where backscattering is minimized, solar energy trapping is enhanced relative to white surfaces, yet thermal emissivity remains controlled.

### 2.2 Realization of White Plasmonic Metasurfaces with Simulation Guider

**Figure 2:**
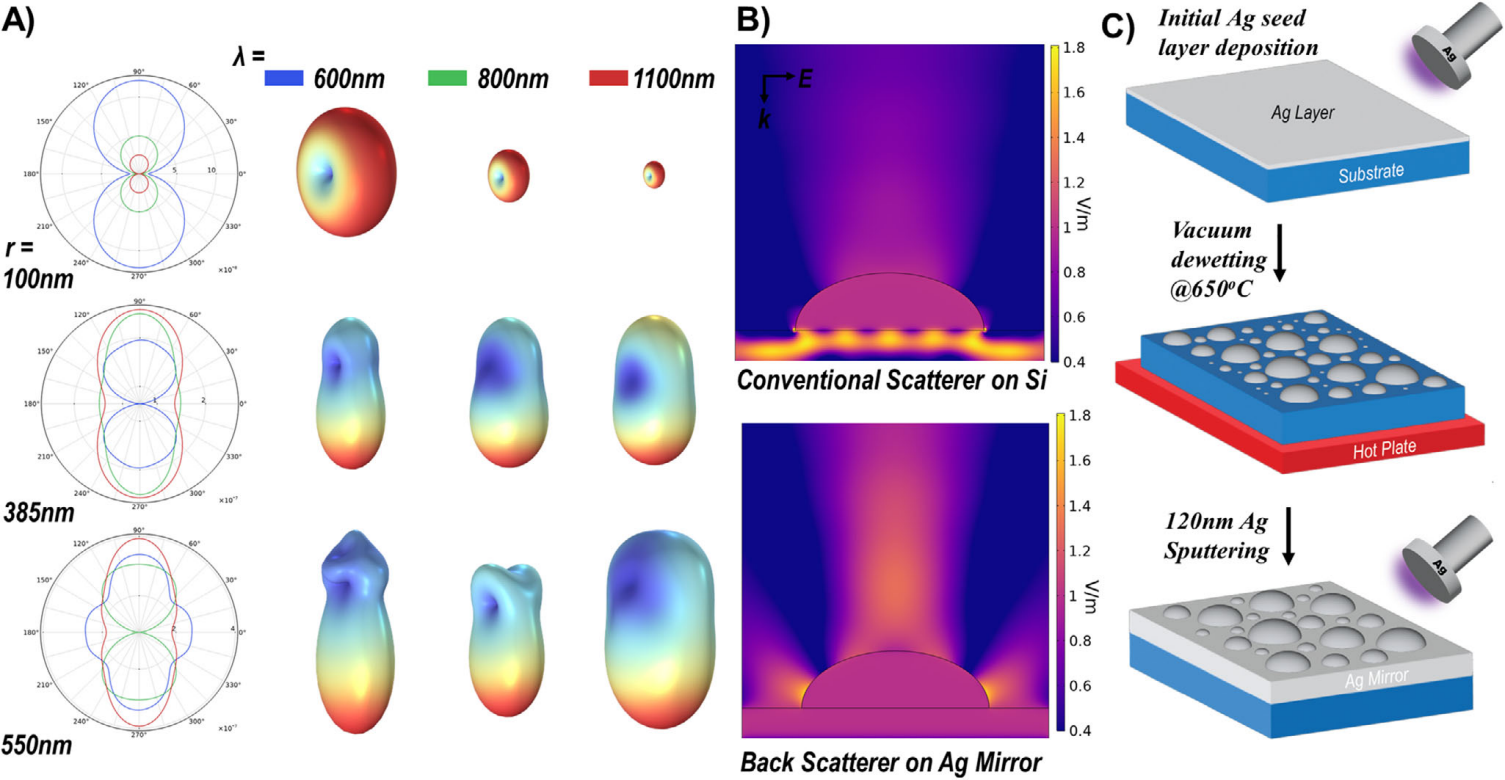
Far‐field scattering characteristics in terms of 2D polar plots 3D scattering profiles of the Silver hemi‐ellipsoids as a function of size and wavelength. B) Optical simulations of the scattering electric field magnitude for an Ag scatterer on a Si substrate and an Ag mirror at 1000 nm excitation wavelength. C) The fabrication process flow of the nanostructured surfaces (NSS) starts by depositing a thin layer of Ag (15, 30, and 50 nm) followed by a solid‐state dewetting step at 650C in a vacuum, lastly and while still in the same device, an optically thick layer of silver is sputtered atop the nanostructures, all under the same vacuum step.

Backscattering is a peculiar phenomenon seen in white colors materials including clouds with high albedo,^[^ ^64^ ^]^ yet replicating it with plasmonic materials is more complex than it seems. Scattering by irregular particles is well understood, with Mie's theory traditionally used to describe such behaviors. However, Mie theory predicts isotropic and/or symmetric scattering for smaller particles up to 100 nm in radius beyond which forward scattering is predominant regardless of particle size or wavelength, as confirmed by our simulations (**Figure** **2**A). Even on substrates, such as irregular particles on silicon, Mie's theory continues to predict forward scattering, resulting in light trapping rather than backscattering, which remains negligible in this configuration (Figure 2B). Interestingly, when silver (Ag) is deposited atop the Ag particles on silicon, backscattering emerges. This occurs because silver's high reflectivity creates a reflective interface, which redirects scattered light back towards its source, resulting in backscattering that can be even tailored according to the size and wavelength as will be shown and discussed later in detail. However, the absence of any trapping on the substrate (Figure 2B) of our configuration highlights the uniqueness of the backscattering mechanisms and reveals new opportunities for advanced light manipulation in solar and thermal spectrums.

Guided by our simulations, we developed a two‐step design approach to systematically induce controlled optical backscattering. Our experimental strategy extends beyond conventional dewetting‐based fabrication, aiming not merely to produce random nanoscale scatterers but to deliberately engineer structures that enhance directional backscattering. Dewetting is a peculiar yet effective process for forming metallic nanostructures;^[^ ^65^, ^66^, ^67^, ^68^, ^69^ ^]^ in our case, it serves as the foundation for a more advanced, simulation‐informed design. The process begins with the deposition of an ultrathin silver (Ag) film onto a clean silicon substrate, followed by vacuum annealing at 650  °C for 50 minutes. This thermal dewetting step yields nano/micro‐island structures with controlled size and distribution. To ensure reproducibility, we maintain strict control over annealing parameters, temperature, duration, and vacuum level, across all fabrication batches. A critical second step involves depositing a thick (120 nm) Ag overlayer. This layer not only enhances mechanical stability but also plays an optical role: it fills interstitial voids, limits further surface migration, and “locks in” the nanostructures, preserving their morphology and scattering characteristics. This structural encapsulation transforms dewetting from a stochastic process into a reproducible method for fabricating surfaces with optimized and consistent backscattering properties. As shown schematically in (Figure 2C) the fabrication begins with the deposition of an ultrathin silver (Ag) layer on a silicon substrate, followed by controlled thermal dewetting through vacuum annealing at 650  °C for 50 minutes. This thermal treatment induces the Ag film to reorganize, resulting in the formation of self‐assembled nano/micro islands. The morphology and distribution of these islands, which are crucial for the metasurface's optical characteristics, depend on the initial layer thickness. This trend is attributed to the quasi‐continuous film formed during initial deposition, where void density depends on thickness as illustrated extensively in the literature. Under vacuum annealing, this quasi‐continuous film reaches a metastable configuration and reorganizes to minimize Gibbs free energy.^[^ ^70^ ^]^

**Figure 3:**
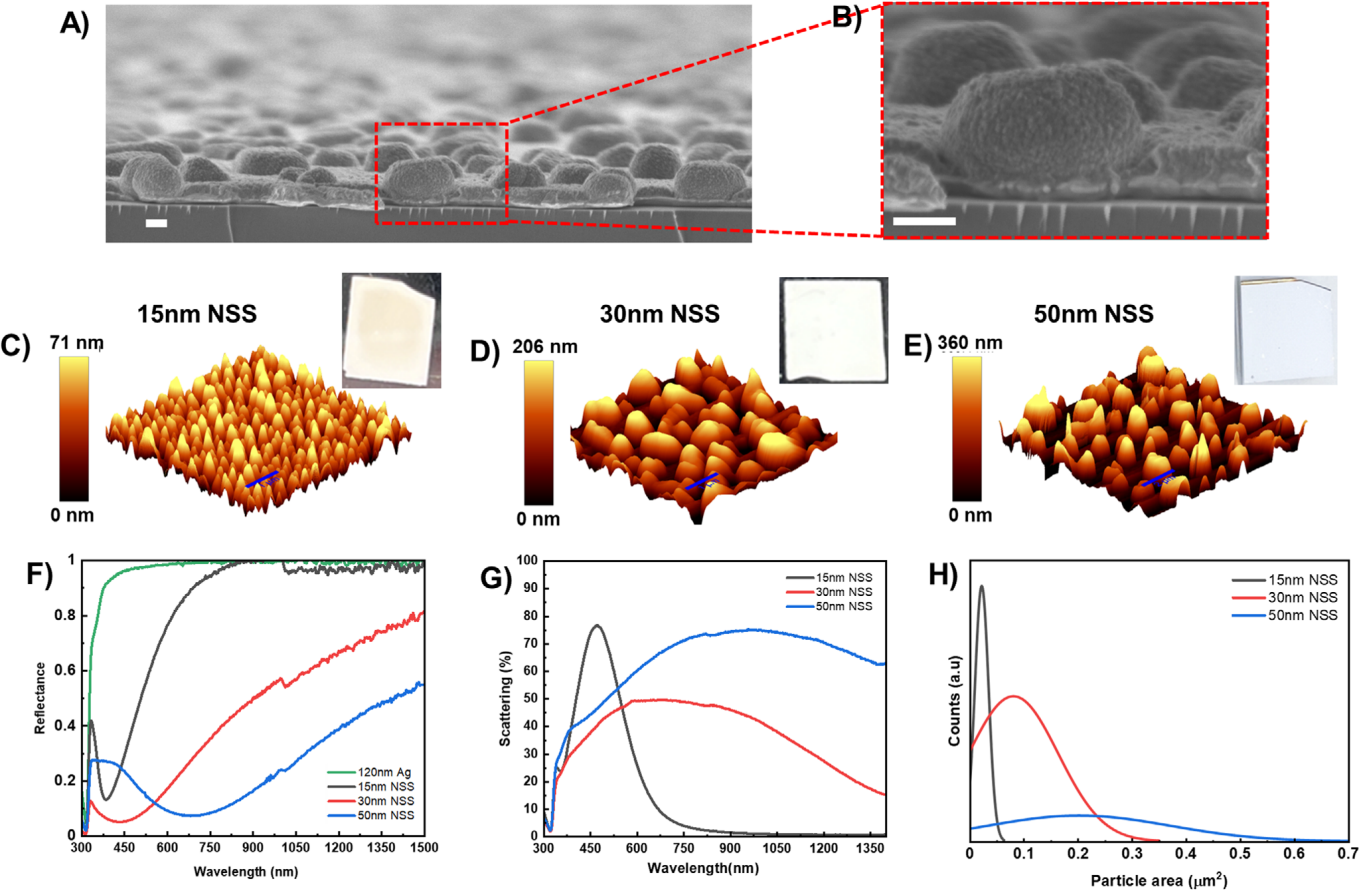
Realization of the plasmonic metasurface, A,B) cross‐sectional SEM images of the 50 nm NSS (Scale bars 200 nm). AFM 3D images of the dewetted Ag initial layer and their corresponding NSS optical images C) 15 nm NSS, D) 30 nm NSS & E) 50 nm NSS with max heights of 71, 206, and 360 nm respectively. F) Reflectance spectra (45° incidence) of the fabricated NSSs compared to an Ag mirror. G) Scattering spectra of the NSSs. H) Particle size distribution of the multiple NSSs fabricated.

The interaction between the Ag layer and silicon substrate surface energies at the annealing temperature prompts the film to transition into a discontinuous state, where nanostructures grow through the nucleation and expansion of voids determined by the original film thickness. After depositing an initial Ag layer and allowing it to undergo dewetting, an optically thick 120 nm Ag film is deposited, completing the metasurface, referred to as the nanostructured surface (NSS). The properties of the NSS are influenced by the thickness of the initial layer, which is set to 15, 30, or 50 nm. The experimental realization and structural configuration of the white plasmonic metasurface is illustrated by the SEM image in (**Figure** **3**A,B), showing the nanostructured surfaces (NSS) with a distinctive disordered arrangement that plays a critical role in its optical properties. These NSSs yield a spectrum of structural properties and resulting color variations as illustrated in (Figure 3C–E) with the white plasmonic effect achieved by using the largest scatterers.

By taking a closer look at the fabrication process and analyzing the AFM images in detail (Figure 3C–E), we were able to measure the surface morphology of the nanostructures. The data shows that as the initial film thickness increases from 15 nm to 50 nm, the nanostructure height grows from 71  to 360 nm, while the overall RMS roughness rises from 17  to 92 nm. This suggests that starting with a thicker layer leads to larger, more varied island formations, which in turn impact the optical performance of the metasurface. A crucial step in the fabrication process involves depositing a thick (120 nm) Ag layer on top of the nano/micro island structures.

**Figure 4:**
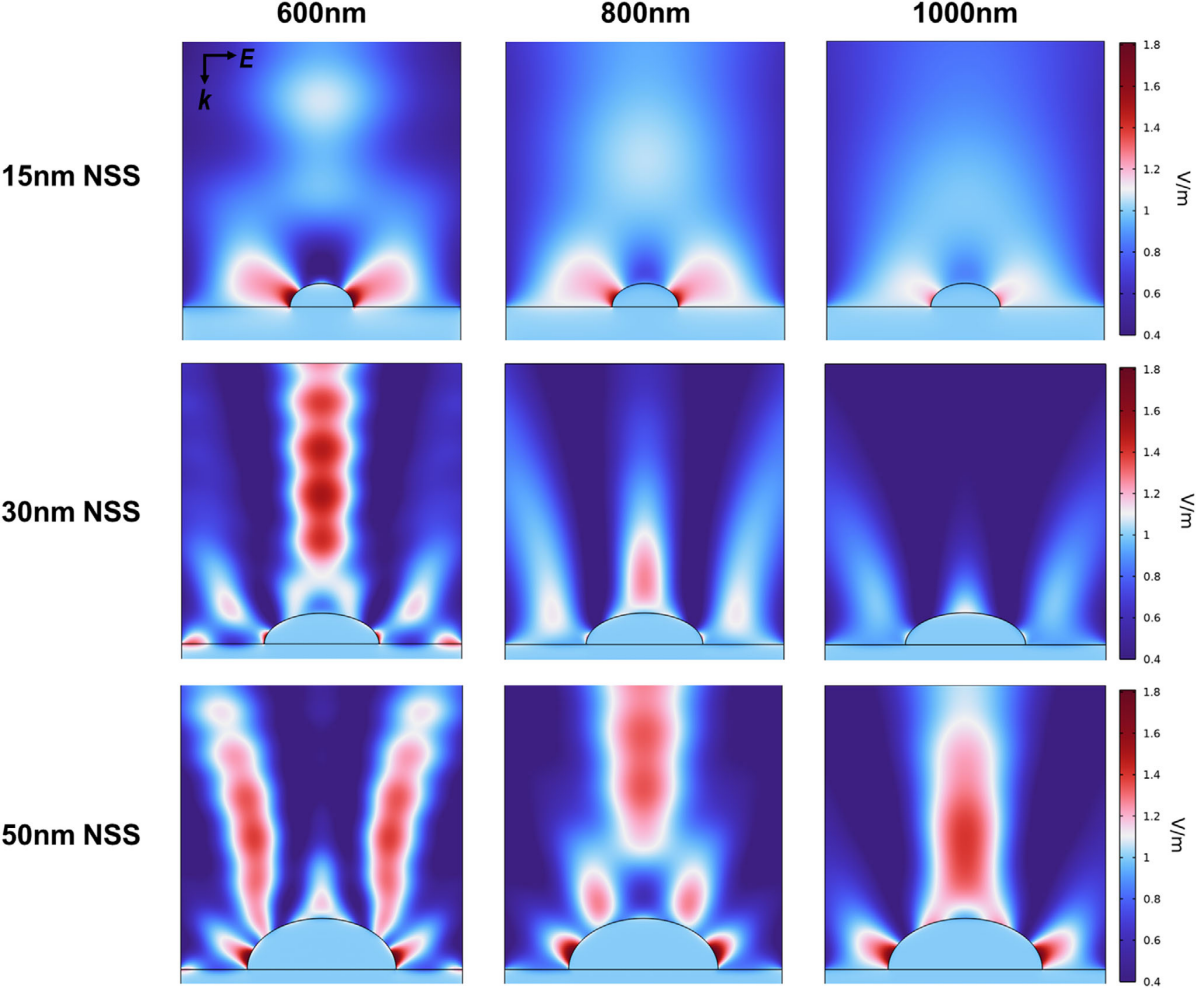
FEM simulations of the scattering electric field magnitude of a silver hemi‐ellipsoid on an Ag mirror as a function of hemi‐ellipsoid size and excitation wavelength.

This additional layer fills voids between the structures, reducing transmittance across the visible and NIR spectral regions and ensuring that light scattering primarily occurs in the backward direction. This structural enhancement differentiates the metasurface from traditional configurations that might promote forward scattering. The enhanced backscattering behavior is critical for the metasurface's properties and is discussed in greater detail later. (Figure 3F) compares the specular reflectance spectra of the fabricated NSSs with that of a standard, flat silver mirror. While the mirror maintains near 100% reflectance throughout the visible and IR range, the NSSs exhibit notable reflectance dips, which have been mostly discussed in the community as an enhanced absorption feature.^[^ ^71^ ^]^ However, measurements using integrated sphere equipment show that these dips correspond to scattering peaks, which shift to longer wavelengths as the nanostructure size increases. These are caused by strong backscattering from the nano/microstructures rather than absorption, confirming the metasurface's unique optical behavior driven by our design. Thus, reflectionless behavior does not inherently equate to absorption. Instead, it arises from enhanced directional backscattering, which primarily contributes to cooling rather than photothermal heating effects. This distinction is important for understanding how our system operates compared to traditional absorptive designs for heating.^[^ ^72^, ^73^ ^]^ For instance, the NSS fabricated with a 15 nm Ag layer presents a sharp reflectance dip at 385 nm, whereas structures made from 30  and 50 nm layers have broader dips at 435  and 690 nm, respectively. This trend is mirrored in the scattering behavior shown in (Figure 3G). The 15 nm NSS exhibits a sharp resonance peak at 460 nm, which shifts to 646  and 946 nm for the 30  and 50 nm structures, respectively. This broadening and redshift are attributed to the increased particle mean area and size distribution (Figure 3H), which introduce multiple resonances from variously sized nanostructures, per Mie scattering theory, along with the diffuse reflection component of the metasurface. By precisely engineering the size and distribution of metallic silver scatterers, we can fine‐tune the backscattering response, transitioning from narrowband backscattering in the visible range to a broadband behavior that extends into the NIR, which is aligned with the simulated scattering cross‐sections as a function of particle size (Figure S2, Supporting Information). Our unique results are confirmed through simulations (**Figure** **4**) demonstrating the influence of scatterer size on the wavelength‐dependent backscattering efficiency. The FEM simulation illustrates the scattered electric field intensities from nanostructured surfaces, modeled with a hemi‐ellipsoid geometry on an optically thick Ag mirror using COMSOL Multiphysics. The simulations reveal that the 15 nm NSS shows a dipolar response with two distinct lobes that diminish in intensity as the wavelength redshifts. In contrast, as the hemi‐ellipsoid size increases, the response shifts to higher resonant modes, forming a robust backscattering profile.

This behavior aligns with our experimental results and underscores the significance of the 50 nm NSS, which exhibits the highest backscattering profile across the selected wavelength range and allows the sample to appear white in color while rejecting the solar spectrum. The experimental validation of this white plasmonic metasurface configuration confirms that it is pivotal for achieving controlled and tunable backscattering spanning the visible and NIR spectra. Smaller scatterers primarily favor backscattering within the visible range, while larger scatterers extend the scattering range, enhancing reflectivity across both the visible and NIR wavelengths. A wide distribution of particle sizes contributes to a broadband scattering profile that covers much of the visible and NIR spectrum, essential for realizing the white plasmonic metasurface. The addition of a metallic layer plays a crucial role in optimizing the backscattering profile. The structural geometry and resulting plasmonic response facilitate a unique mechanism that leverages radiative and backscattering processes, setting this design apart from conventional approaches.

### 2.3 White‐to‐Grey Plasmonic: Hidden Thermal Signature with Cooling and Heating

Disordered plasmonic and refractory metal metasurfaces have been previously explored for broadband absorption and black coloration for heating applications, driven by their randomized resonant modes.^[^ ^22^, ^74^, ^75^ ^]^ In contrast, the concept of achieving plasmonic grey coloration from white surfaces through innovative material structural designs, particularly those that do not rely on conventional pigmentation methods, has not been thoroughly explored in the scientific literature. In pigmentation theory, grey is typically achieved by mixing black and white in equal proportions, resulting in a neutral color without hue. This principle is based on subtractive color mixing, which applies to pigments and physical media like paint. However, the optical phenomena of grey clouds offer a different perspective. Unlike traditional concepts of pigmentation color mixing, clouds, and aerosols interact in unique ways to influence perceived colors. In nature, aerosol layers can cause clouds to appear grey by modifying how sunlight interacts with the cloud. The inherent white scattering of the cloud is partially “diluted” by light‐absorbing aerosols, preventing full absorption and the appearance of pure black. Instead, a grey color emerges due to the interplay between scattering cancellation and partial light absorption. This phenomenon provides a blueprint for designing advanced absorbers and sustainable grey materials. Mimicking the cloud‐aerosol interplay, we achieve a similar white‐to‐grey color transition through the interaction between a white NSS metasurface and nanocomposite layer, which acts as the “aerosol.” This design leverages light manipulation principles, moving beyond traditional pigmentation rules to create tunable, grey‐color materials inspired by nature.

**Figure 5:**
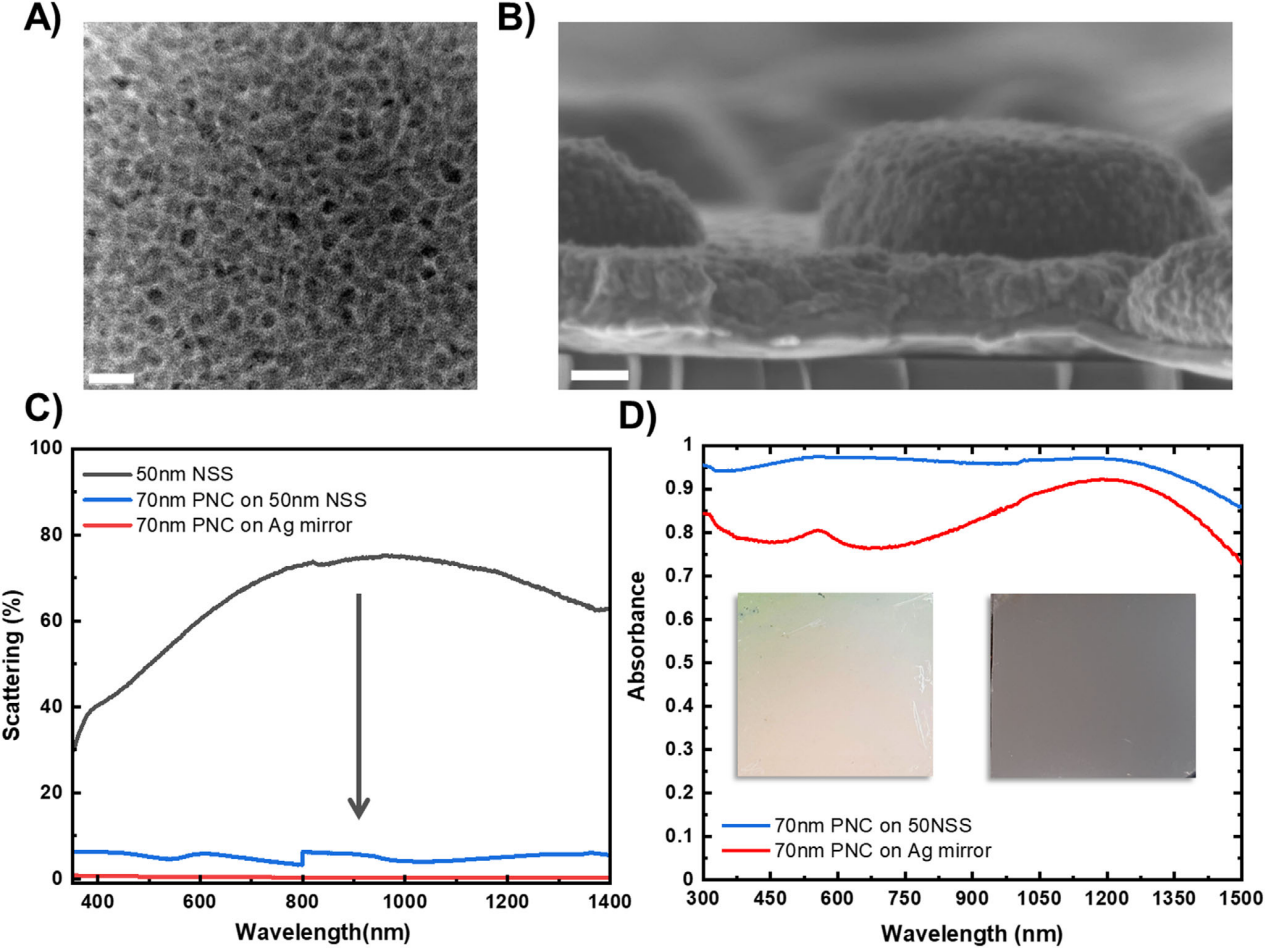
A) HRTEM image (scale bar 10 nm) of a 48% filling factor (FF) Cu/Al2O3 plasmonic nanocomposite (PNC). B) Cross‐sectional SEM image of a grey metasurface with a 70 nm PNC atop a 50 nm nanostructured surface (NSS) on a silicon substrate (scale bar 100 nm). C) Scattering spectra comparing a 50 NSS before and after the deposition of a 70 nm PNC layer, as well as atop an Ag mirror. D) Absorbance spectra of a 70 nm PNC layer atop a 50NSS and an Ag mirror. Inset) optical images of the 70 nm PNC layer atop the Ag mirror (left) and NSS (right).

For the design of the absorber layer, a plasmonic nanocomposite (PNC), described in detail elsewhere,^[^ ^50^, ^76^ ^]^ was deposited atop a 50 nm NSS layer, which corresponded to the whitest and most broadband scattering. The PNC consists of randomly distributed Cu nanoparticles (2r≈3 nm) embedded in an Al₂O₃ matrix, with a metallic filling factor (FF%) of 48% Cu, as shown via TEM (**Figure** **5**A). The selected nanocomposites act as an absorbing and reflecting polarizonic medium that is active in visible and NIR with minimized scattering (Figure S3, Supporting Information). The structural configuration of the NSS metasurface ensemble, prior to nanocomposite deposition, is revealed in the cross‐sectional SEM image (Figure 5B). The image displays a 70 nm PNC layer atop the 50 nm NSS layer on a silicon substrate, where no major deviation in structure was noticed. Notably, complete scattering cancellation (Figure 5C) from UV to NIR is observed in the white silver 50 nm NSS sample when the 70 nm PNC layer has been deposited on top. Similarly minimum scattering is also expected from the 70 nm PNC layer atop Ag mirror as neither constituent possesses scattering properties. The broadband scattering of the 50 nm NSS, accompanied by broad absorption (Figure 5D) across the same spectral range, results in the grey appearance of the sample, an outcome unattainable with metallic silver alone that gives rise to green‐reddish coating, as seen in the optical images as figures inset. In optical theory, when white light, comprising all visible wavelengths, interacts with black, which absorbs all light, the result is the absence of reflected light, producing a black appearance. In our case, although the near‐complete absorption (97%) across a broad wavelength range of 250  to 1500 nm yields a grey appearance, and not black. This phenomenon is experimentally realized here for the first time. The grey color arises from a meticulously engineered balance of scattering cancellation, light trapping, and absorption within the layered structures, blending the principles of optical light manipulation and pigmentation. This is the first experimental demonstration of materials mimicking the grey cloud principle with near‐matching RGB color coordinates (Figure S7, Supporting Information), providing novel insights into its mechanism and opening pathways for advanced light manipulation and energy harvesting applications.

**Figure 6:**
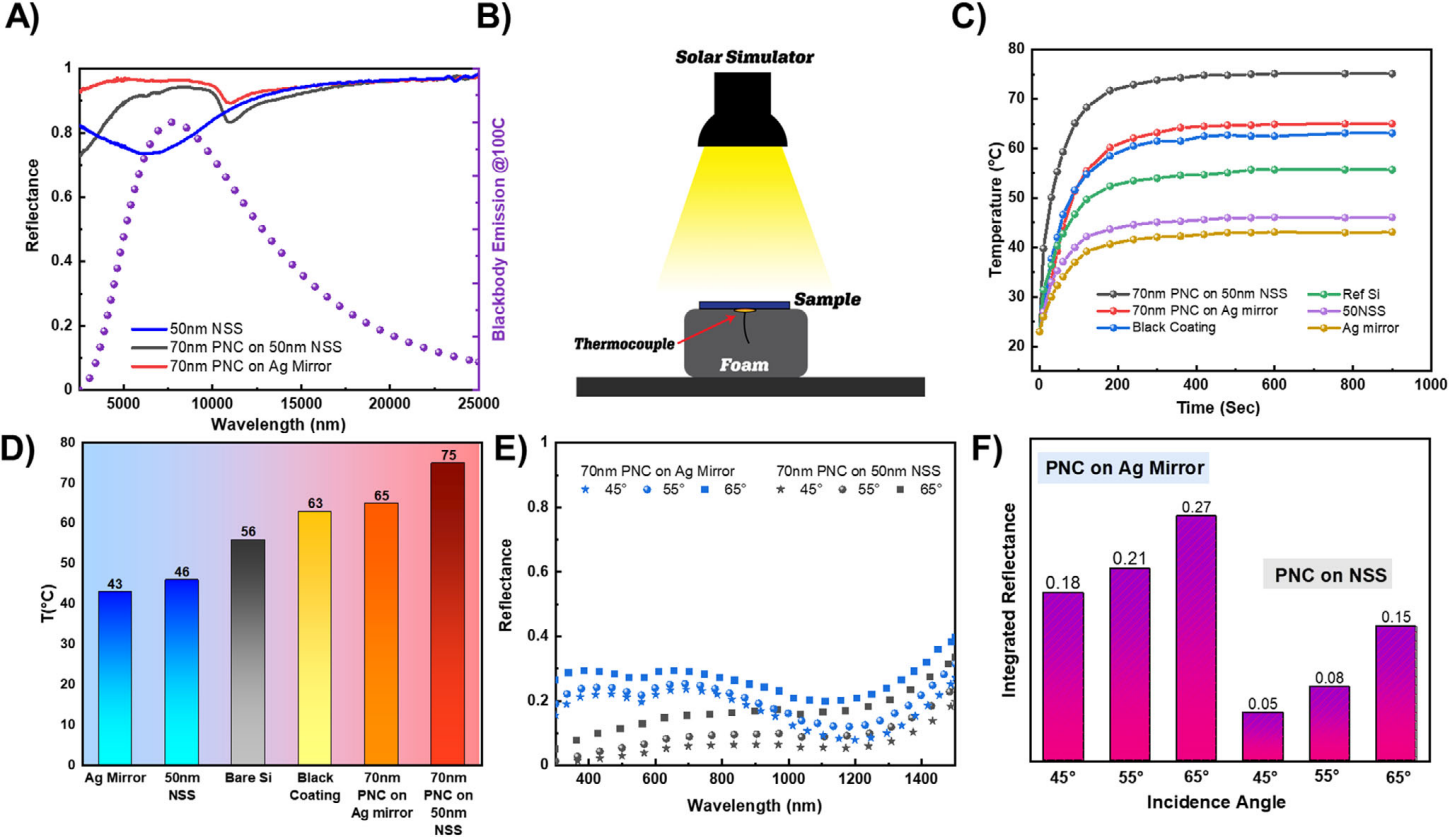
A) IR reflectance spectra of the fabricated structures compared to the blackbody thermal radiation at 100 °C. B) Sketch depicting how the sample temperature was measured. Measured C) time series and D) steady‐state temperatures obtained from exposing the samples to 1‐sun radiation. The measured temperatures under 1‐sun for multiple samples. E) Measured reflection spectra at multiple incidence angles of the 70 nm PNC atop a mirror (blue), and 50 nm NSS (gray). F) The histogram show the integrated reflectance values of both absorbers at different angles of incidence.

The metasurfaces not only resemble white and grey clouds in appearance but also replicate their functional properties. Just as clouds obscure the Earth from satellites, these fabricated metasurfaces, with their white and grey coloration, exhibit similar camouflage capabilities with hidden thermal signatures. As shown earlier in (Figure 1), the white metasurface seamlessly integrates into cloudy and snowy landscapes, while the grey metasurface mimics aerosol‐laden clouds, providing light trapping and heating functionalities. These attributes are further enhanced by the thermal concealment provided by the metasurfaces' infrared reflectivity, ensuring low emissivity for both the white and grey samples. This property allows the metasurfaces to effectively obscure systems with thermal signatures, including those operating at 100 °C, as demonstrated by the low emissivity observed in (**Figure** **6**A). The calculated emissivities (e = 1 – R) for the 50 nm NSS, 70 nm PNC on the Ag mirror, and 70 nm PNC atop 50 nm NSS are 0.11, 0.04, and 0.08, respectively. It is important to note that the features appearing in the curve are attributed to the Al‐O bond arising from the PNC matrix and scattering due to the nanostructures. The combination of white/grey plasmonic colorations with low emissivity highlights the distinctiveness of our metasurfaces, a capability not achievable by traditional white or black metal oxides or organic and polymeric materials. This functionality is particularly important for applications requiring optical camouflage and thermal stealth, such as in military or wildlife contexts. In addition to optical camouflage and thermal concealment, these metasurfaces enable dynamic temperature regulation. Low‐emissivity (low‐e) materials have been widely used to reduce energy consumption in both opaque and transparent building areas by limiting heat transfer via thermal radiation.^[^ ^77^ ^]^ However, our focus here is to introduce local cooling management at the substrate. Indeed, the white metasurface reduced the temperature of a silicon substrate by a relative −10 °C under 1‐sun radiation, acting as an effective cooling layer (Figure 6C,D). Unlike conventional passive cooling systems, which target ambient temperature reduction, our method prevents heat buildup directly at the substrate level, ensuring superior performance even under intense solar illumination. This innovation provides a more efficient cooling solution and enhances thermal control in applications such as photovoltaics and microelectronics, where localized cooling is essential. Similarly, our white metasurface surpasses their conventional white coatings in functionality. Conventional white coatings, being bulk materials, suffer from high infrared emissivity and thus fail to manage substrate thermal loads effectively, making them unsuitable for thermal camouflage. In contrast, our white metasurface is engineered for low emissivity in the thermal IR, enabling substrate cooling while remaining undetectable by thermal cameras. Notably, the cooling performance of the low‐e white metasurface was comparable to that of an Ag mirror, maintaining similar heat rejection but with the added advantages, such as a camouflaged white appearance and its novelty as a substrate capable of designing a perfect solar absorber both not attenable by the mirror counterpart, as demonstrated in the previous section. Unlike conventional systems, where white and black components are fundamentally different materials with distinct cooling and heating properties, our metasurface seamlessly integrates a white state with a switchable gray absorber state on a single template. This dual‐state functionality provides both efficient substrate cooling through high backscattering and controlled heating via broadband absorption, overcoming the limitations of traditional coatings that rely on separate materials for these functions. As also shown in (Figure 6B,C), the white‐driven grey configuration is a unique solar‐thermal converter design that achieved a significantly higher stable temperature of 74 °C, surpassing its planar plasmonic counterpart (65 °C) and the black‐based absorbers (63 °C). This higher performance originates from the high scattering of the substrate, which traps the absorbed light within the absorber layer, mimicking the behavior of grey clouds. Indeed, our grey‐colored absorber not only achieves effective broadband absorption but also outperforms conventional black absorbers and even plasmonic absorbers on metal by delivering up to a relative +10 °C improvement in thermal management gain reinforcing the superiority of our design for dynamic thermal control.

Another key property of solar absorbers is the ability to maintain high absorbance across a broad range of wavelengths, from 250  to 1500 nm, and at various incident angles, such as those during sunrise and sunset. This is particularly important for locations at latitudes far from the equator. The angle‐dependent reflectance data in (Figure 6E,F) show that the NSS consistently outperforms the planar absorber at all angles of incidence and polarizations (Figures S5, Supporting Information), maintaining a grey color with an omnidirectional response. For example, at 45°, the NSS absorber achieved an average solar absorption of 0.95, compared to 0.82 for the planar absorber. This result is expected, as the metasurfaces scatter light in all directions due to the random orientation of the nanostructures, resulting in nearly omnidirectional absorption behavior when the PNC layer is overlaid. Lastly, the stability of the metasurfaces was evaluated after two years of storage (Figure S6, Supporting Information), revealing that the white sample experienced a slight redshift and a ∼10% decrease in scattering efficiency, but its optical performance remained robust. The grey sample showed minimal changes in optical behavior, demonstrating the effectiveness of the PNC layer in protecting the NSS and highlighting the metasurfaces' practical stability for consumer applications.

## 3 Conclusion

This study presents a groundbreaking advancement in the design of plasmonic metasurfaces, inspired by the natural mechanisms of cloud‐aerosol interactions, to control both the visual and thermal properties of materials. By engineering the first‐ever plasmonic disordered metasurfaces to exhibit white and grey color effects, this approach offers a deeper understanding of the radiative forcing of cloud‐aerosols with complicated optical properties and provides practical insights for the design and development of superior heat management, making it ideal for energy‐efficient applications. Our study reveals that achieving efficient cooling and thermal camouflage often requires materials that can provide significant backscattering in the solar spectrum combined with low emissivity in the thermal infrared range. Opposite of the current direction of radiative cooling that requires diffuse scattering in the solar spectrum combined with high emissivity in the thermal infrared range. Through simulations and experimental validation, we demonstrated that the white metasurface effectively mimics the clouds by backscattering solar radiation, achieving a relative −10 °C substrate temperature reduction under 1‐sun illumination. Our finding surpasses the capabilities of traditional passive cooling methods by shifting the focus from environmental cooling to targeted substrate temperature management. The study also underscores the critical role of emissivity in thermal management. Low‐emissivity materials like white silver offer superior performance in both cooling and heating contexts by minimizing heat loss through radiation. This contrasts with highly emissive white paints, which, despite their reflective properties, tend to retain heat inefficiently. The key innovation is the design of a white metasurface that transforms into grey through an engineered process. By incorporating a nanocomposite absorber, this transformation relies on backscattering cancellation, light trapping, and absorption, resulting in grey coloration not achievable with metallic surfaces. For the first time, we demonstrate near‐complete absorption (97%) across a broad spectral range (250  to 1500 nm), driven by advanced optical manipulation rather than pigmentation. This omnidirectional grey configuration boosts heating performance, increasing temperatures by a relative +10 °C compared to conventional absorbers and even surpasses black surface. Taken together, our findings introduce a new class of white plasmonic substrate materials that offer exciting opportunities for adaptive surfaces, energy‐efficient coatings, and advanced thermal regulation. Furthermore, the ability to control thermal signatures and provide camouflage for various applications, ranging from military stealth to energy‐efficient technologies, marks a significant step forward in the field. The research not only highlights the potential of cloud and aerosol‐inspired plasmonic systems but also opens the door to future innovations in dynamic, multifunctional materials for thermal management and visual concealment. Our all‐in chamber nanofabrication design enables future sustainable research while avoiding organic and/ or inorganic pigments with upscaling opportunities relaying on sputtering technology, paving the way for their integration into real‐world energy and thermal management applications.

## 4 Experimental Section

The Si wafer substrate utilized in this study is a p‐doped Si wafer, with (100)‐orientation and a native oxide layer. The fabrication process of the samples in this work was done using a custom‐designed and assembled magnetron sputtering apparatus which consists of two magnetrons that are operated independently, and a sample holder equipped with a rotatable motor to ensure uniform film deposition and prevent composition and thickness gradients. The Ag nanostructures were obtained by first depositing an initial Ag film with thicknesses of either 15, 30, and 50 nm using DC sputtering at room temperature. Subsequently, these initials films underwent annealing at elevated temperatures of 650 °C at a rate of 30 °C min^−1^ and were kept at the target temperature for 50 minutes on both silicon and glass substrates. After the samples were cooled to room temperature, a 120 nm thick Ag mirror using DC sputtering was deposited atop the annealed initial films forming the nanostructured surfaces NSS, where the prefix refers to the initial layer thickness. For the fabrication of the plasmonic nanocomposite (PNC) co‐deposition technique was utilized where the Al2O3 dielectric and copper components were sputtered using RF and DC magnetron sources respectively. Both sources were positioned at a 50° angle relative to the substrate and placed in opposite directions relative to the sample holder. The filling factor of the composite was determined based on the deposition rates of each component under the same gas flow rate and pressure conditions used for co‐sputtering. The optical properties of the fabricated samples were determined using a spectroscopic ellipsometer (M2000‐UI, J.A. Woollam) by measuring the reflectivity of the samples over a spectral range of 245  to 1690 nm. The ellipsometer data was recorded and controlled using the CompleteEase software (J.A. Woollam). The study considered the entire sample as optically thick, thus neglecting light transmission. The reflectance spectra in the range 2500–25 000 nm were measured by Alpha II ATR FT‐IR spectrometer (Bruker). The surface topography was measured using an atomic force microscope AFM (alpha300 RA‐Raman‐AFM‐Microscope, WITec) with an AC240 TS AFM tip (Asylum Research) to characterize the surface topography. Areas of 5 × 5 µm were measured in 512 × 512 points or 10 × 10 µm were measured in 1024 × 1024 points, depending on the particle size. WITec software was used to determine the RMS roughness. The morphology was analyzed using scanning electron microscopy (SEM) Gemini Ultra 55 (Zeiss, Germany) in top‐ and side‐view configurations for EDS measurements Oxford instruments apparatus was used. Bright‐field images were acquired using a Transmission electron microscopy (TEM) Tecnai F30 G2 (FEI) operating at 300 kV to visualize the nanocomposite's particle size distribution. The nanocomposite was directly deposited on a TEM grid (Ni mesh 300 with C film, Plano EM, S160N3). Solar‐thermal conversion was measured using a calibrated solar simulator equipped with a 1.5 air mass filter (ABET Sunlite) at 1 sun (1000Wm^−2^) direct incidence. The temperature was recorded after 15 mins of exposure via a k‐type thermocouple attached to the samples back. FEM simulations were obtained via COMSOL Multiphysics where the silver's imaginary part of the optical constant was multiplied by 1.5 to account for size‐induced damping effects. The sizes of the hemi‐ellipsoids in terms of radius1 x radius2 x height are 200 × 120 × 71 nm, 770 × 550 × 206 nm, and 1100 × 660 × 360 nm for the 15, 30, and 50 nm initial film, respectively. For the 3D far field calculations and their respective 2D polar plots, a 3‐D model compromising of hemi‐ellipsoids whose dimensions were determined from the experimental data was surrounded by a spherical PML and subjected to a plane wave. Whereas for the scattered electric field magnitude, a 2D model compromising of the same hemi‐ellipsoids atop an optical thick Ag mirror and surrounded by square PML was subjected to a plane wave propagating in the y‐direction with an oscillating electric field in the x‐direction.

## Conflict of Interest

The authors declare no conflict of interest.

## Supporting information



Supporting Information

## Data Availability

The data that support the findings of this study are available from the corresponding author upon reasonable request.
