# Supplementary material for: Cloud Inspired White and Grey Plasmonic Metasurfaces for Camouflaged Thermal Management
Source: Adv Mater. 2025 Jun 6;37(35):2501080. doi: 10.1002/adma.202501080 (PMC12412007; doi:10.1002/adma.202501080)
Supplement: Supplementary file 1 — Supporting Information [file ADMA-37-2501080-s001.docx]

Supporting Information

Cloud Inspired White and Grey Plasmonic Metasurfaces for Camouflaged Thermal Management

Mhd Adel Assad^1^, Moheb Abdelaziz^1,2^*, Torge Hartig^2^, Thomas Strunskus^2^, Alexander Vahl^2^, Franz Faupel^2^, Mady Elbahri^1,2*^

^1^ Nanochemistry and Nanoengineering, School of Chemical Engineering, Department of Chemistry and Materials Science, Aalto University, Finland.
E-mail: Mady.Elbahri@aalto.fi

^2^ Institute for Materials Science, Chair for Multicomponent Materials, Faculty of Engineering, Kiel University, Kiel, Germany.

| 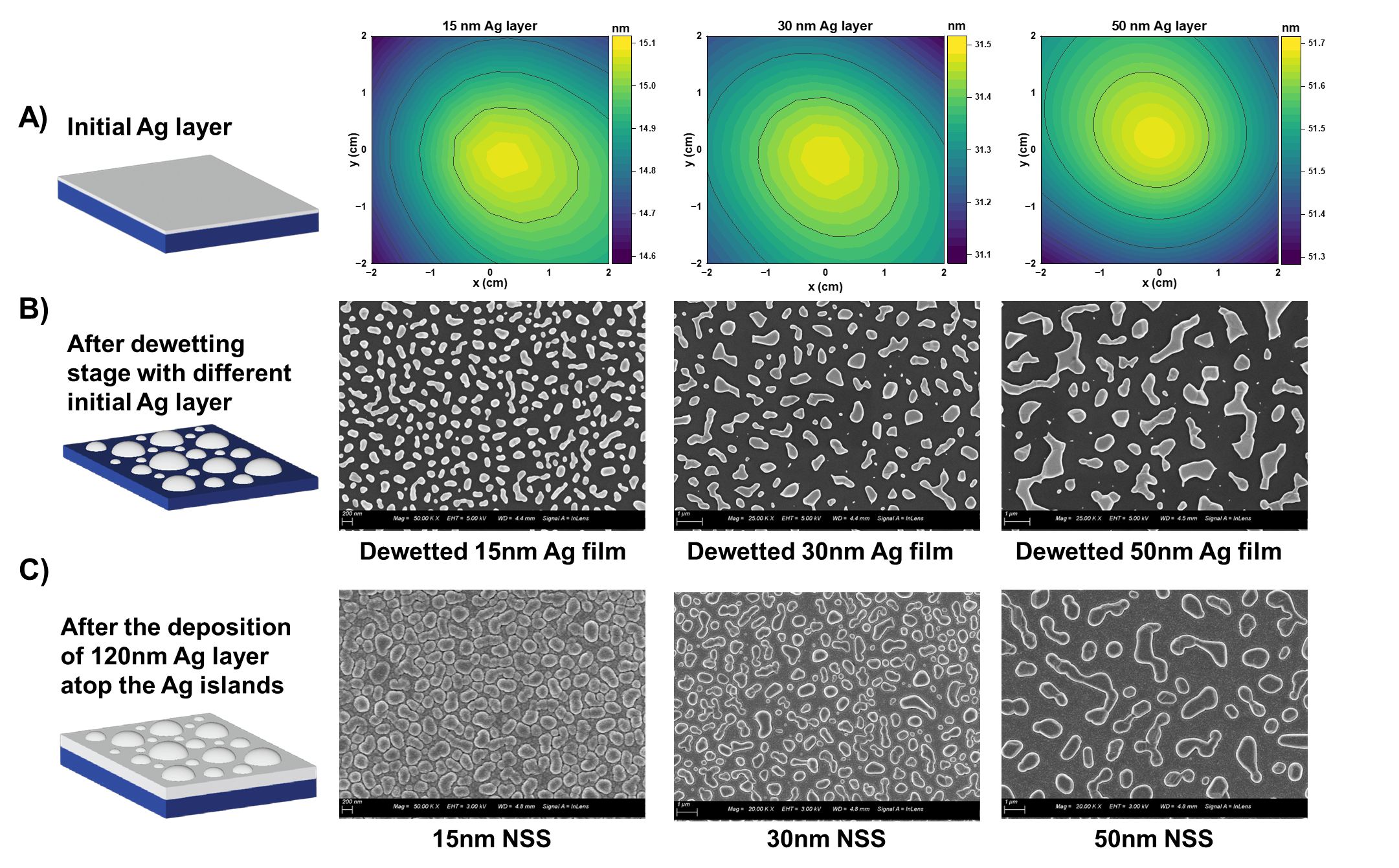 |
| --- |
| Figure S1. Fabrication process and nano/micro islands formation. A) contour plots of the thickness distribution across a 2x2 cm^2^ sample showing minimum gradient effects for the initial silver films. B) SEM images of the dewetted initial Ag layer forming island with increasing size depending on the initial layer thickness. C) SEM images of the nanostructured surfaces NSS which is formed after the deposition of a mirror-thick Ag layer atop the dewetted structure. |

The fabrication process of the nanostructured surfaces (NSS) is carried out entirely within a single device and under a single vacuum step, ensuring a controlled and contamination-free environment. It begins with the deposition of an initial silver (Ag) layer, which serves as a seed layer for the formation of nano- and micro-scale islands. In this work, we selected initial layer thicknesses of 15 nm, 30 nm, and 50 nm. As shown in (**Figure S1 A)**, the thin films exhibit high conformality with minimal gradient effects. After depositing the initial layer, and while still in vacuum, the substrate is heated to 650°C for 50 minutes using a built-in heating stage. This step initiates a dewetting process, transforming the conformal thin film into discrete islands driven by the surface energy of silver, as observed in the SEM images in (**Figure S1 B)**. The initial layer thickness plays a critical role in determining the island sizes and their distribution, providing precise control over the nanostructure formation. Once the substrates return to room temperature, a 120 nm thick Ag mirror is deposited atop the structures, completing the transformation into what we refer to as the NSS metasurface, as illustrated in (**Figure S1 C**).

| 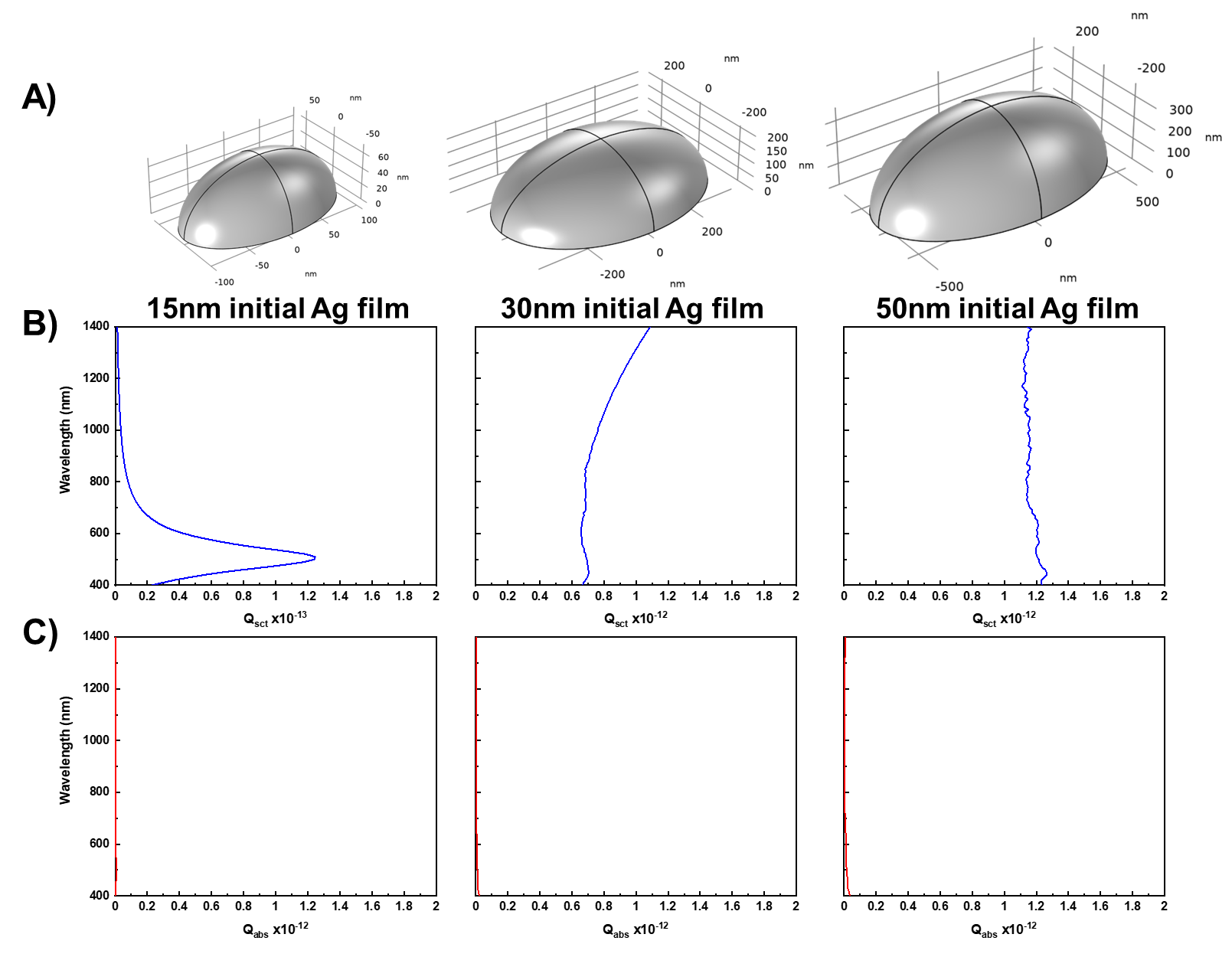 |
| --- |
| Figure S2. Hemi-ellipsoids optical properties. A) geometrical sizes used for the FEM study of the silver hemi-ellipsoid obtained from the SEM and AFM data. Calculated B) Scattering and C) Absorption cross-sections of Hemi-ellipsoids as a function of size. |

Using COMSOL, the scattering and absorption cross-sections of the selected hemi-ellipsoids were analyzed. The dimensions of these hemi-ellipsoids are depicted in (**Figure S2 A)**, where it is shown that as the initial Ag layer thickness increases, the particle size also increases. These dimensions were obtained from SEM and AFM analyses, as presented in (**Figure S1)** and discussed in the manuscript, which in terms of radius1 x radius2 x height are 200x120x71 nm, 770x550x206 nm, and 1100x660x360nm for the 15, 30, and 50 nm initial film, respectively. The simulation results align closely with experimental observations. For an initial Ag layer of 15 nm, the scattering peak appears in the visible range, consistent with experimental data, whereas particles formed from 30 nm and 50 nm initial layers exhibit broadband scattering with increasing intensity as particle size increases (**Figure S2 B)**. In all cases, absorption cross-sections remain minimal (**Figure S2 C)**, confirming that scattering is the dominant contributor to particle extinction.

| 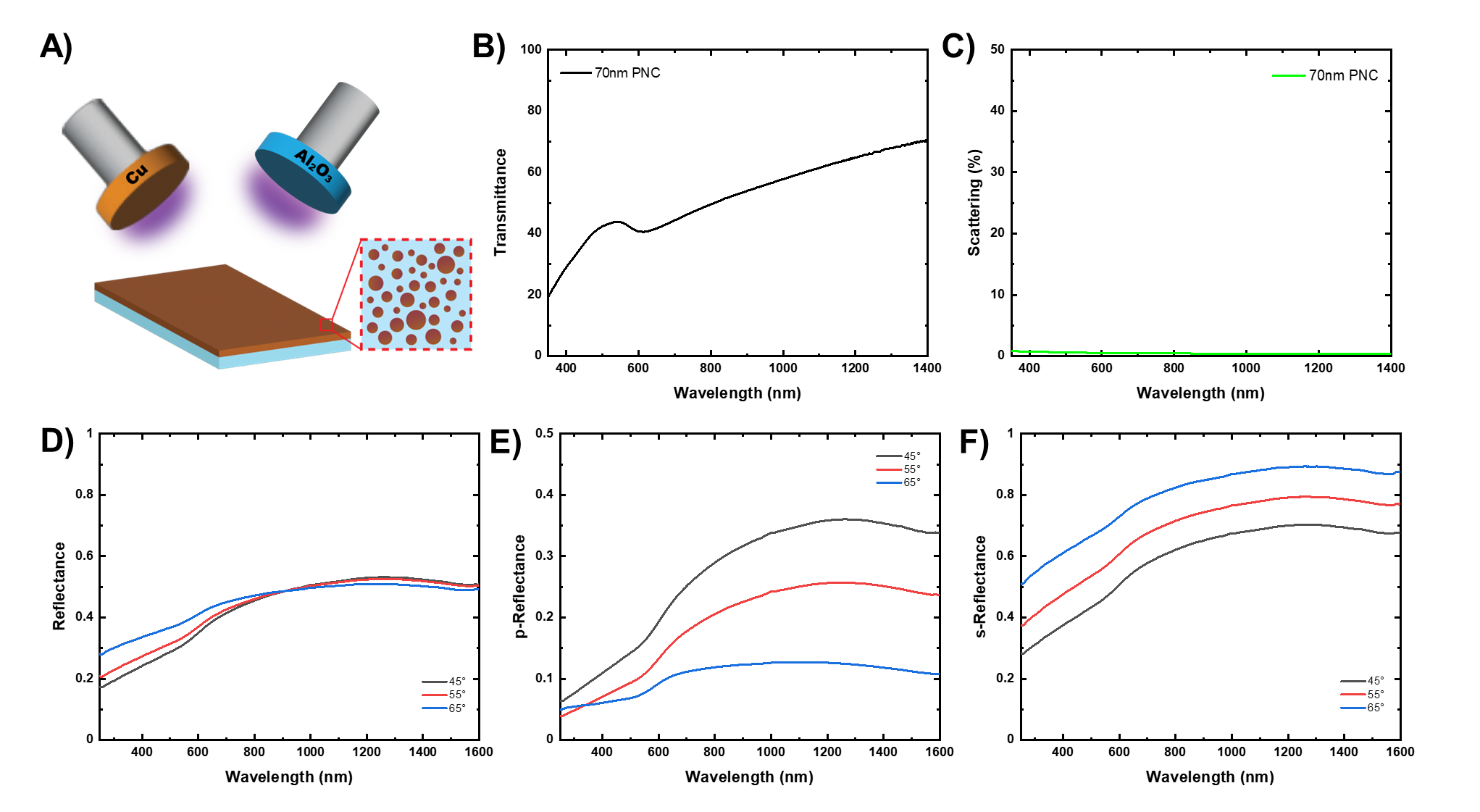 |
| --- |
| Figure S3. Plasmonic nanocomposite (PNC) fabrication and optical performance. A) schematic depicting the fabrication procedure by co-deposition of Copper and Alumina for the formation of a nanocomposite absorber. B) Transmission, C) Scattering, D) Total reflection, E) P-reflection, and F) S-reflection of the 70nm PNC on a glass substrate. |

The fabrication process of the plasmonic nanocomposite (PNC) is illustrated in (**Figure S3 A**). This process involves the co-deposition of a metal, in this case, copper, and a dielectric, alumina, resulting in the formation of a segregated-phase composite. The nanoparticles, driven by surface energy, are randomly dispersed within the alumina matrix, which prevents agglomeration and oxidation. This nanocomposite functions as a light-absorbing medium, covering the UV to NIR regions of the spectrum (**Figure S3 B**). The kink observed around 600 nm is attributed to the localized surface plasmon resonance (LSPR) of the copper nanoparticles. Despite the nanoparticles being ultrafine, as evidenced by the TEM images in the manuscript, they do not exhibit any significant scattering properties (**Figure S3 C**). The PNC shows a polarizonic character ^[1,2]^ observed in its reflection spectra in NIR (**Figure S3 D-F**) while matching with the backscattering of the white metasurface. The film's reflectance increases with the incidence angle, as expected. Moreover, with p-polarized light, the reflectance decreases as the incidence angle, highlighting the Brewster polarizonic behavior of the PNC layer ^[1,2]^. Similar to the transmission spectra, all reflectance spectra show the LSPR dip at 600nm, shows the absorption character of the nanocomposite.

| 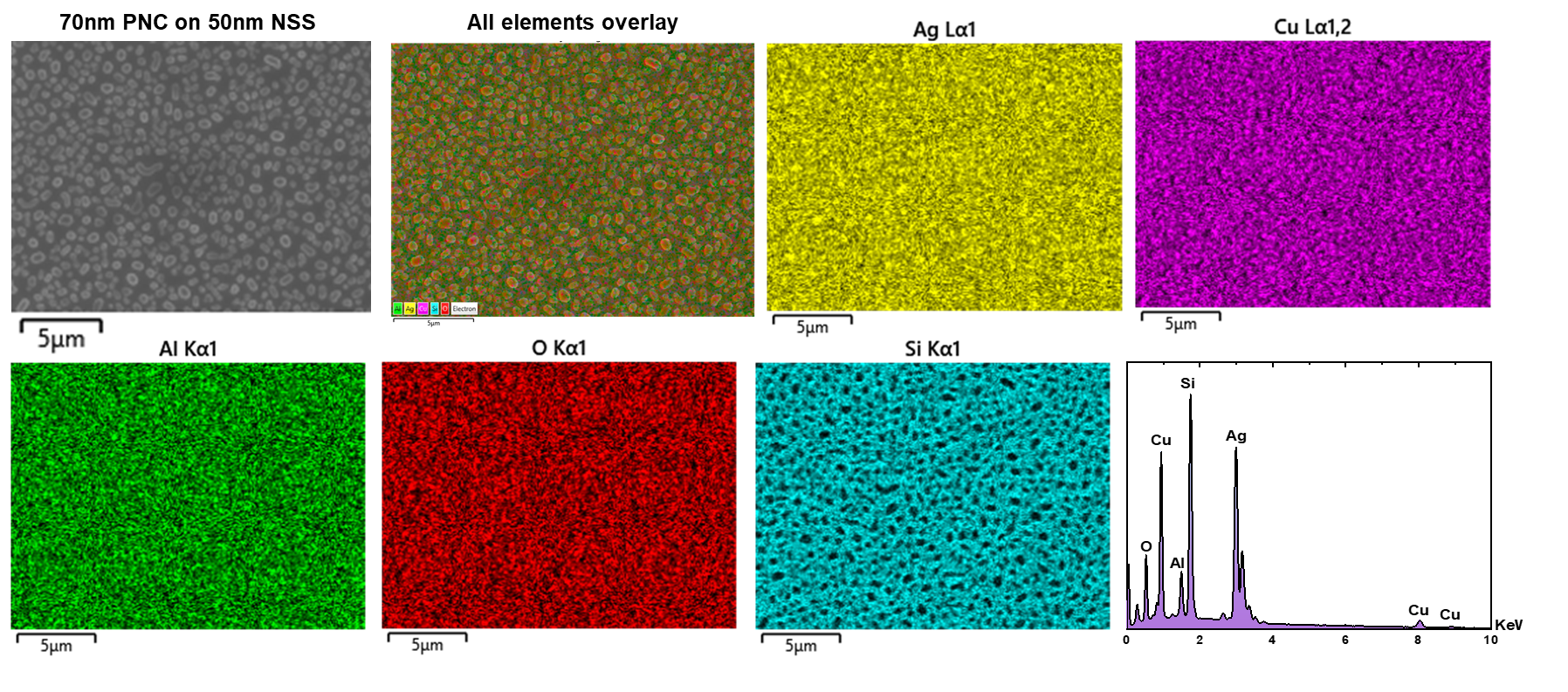 |
| --- |
| Figure S4. Energy Dispersive Spectroscopy (EDS) analysis of a 70nm PNC deposited atop a 50nm NSS showcasing the uniformity of deposition across the sample. |

Energy Dispersive Spectroscopy (EDS) analysis was performed on the grey sample, comprising a 70 nm plasmonic nanocomposite (PNC) layer atop a 50 nm nanostructured surface (NSS). The results revealed a homogeneous distribution of all constituent elements in the metasurface structure: silver (Ag) from the NSS acting as a structured mirror surface, copper (Cu) nanoparticles, and aluminum (Al) and oxygen (O) from the alumina matrix in the PNC layer. Additionally, silicon (Si) from the underlying substrate was detected. Notably, the Si elemental map exhibited dark zones corresponding to areas with increased Ag thickness, corroborating the presence of nano/micro islands observed in the AFM analysis.

| 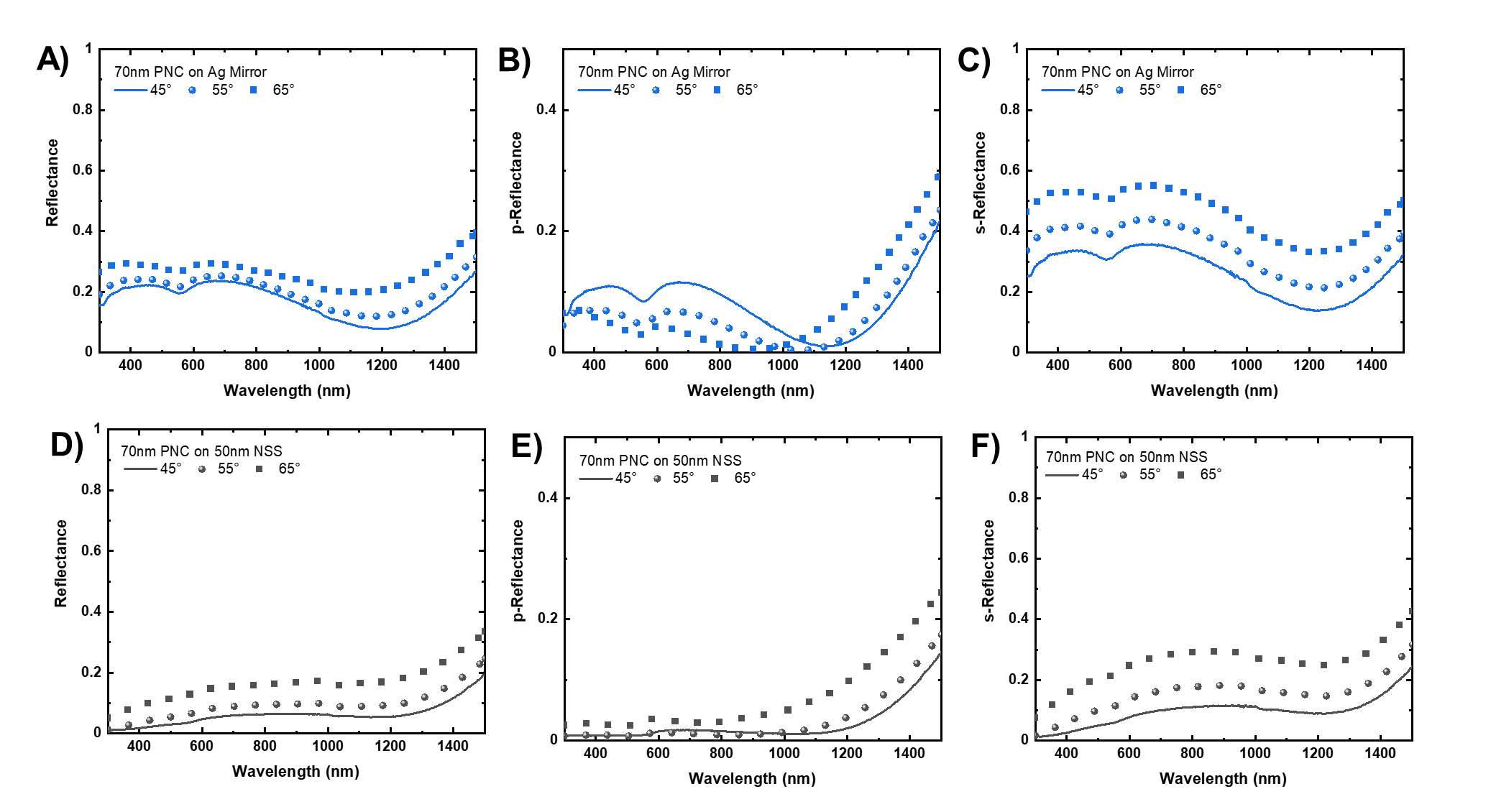 |
| --- |
| Figure S5: Optical multiangle and polarization response of the 70nm PNC atop Ag mirror in terms of A) total, B) p-, and C) s- reflectance. Similarly, for a 70nm atop 50nm NSS in terms of D) total, E) p-, and F) s- reflectance. |

A comprehensive optical analysis was conducted to compare the performance of absorbers on planer surfaces versus those atop nanostructured surfaces (NSS) across various incidence angles (45°–65°) and polarizations (s- and p-) (**Figure S5**). The findings indicate that NSS-based absorbers consistently outperform their planar counterparts across all tested angles and polarizations. Typically, an increase in reflectance is observed as the incidence angle increases. However, in the p-reflection spectra of the planer-based absorber (**Figure S5 B**), a blue-shifting dip is noted, attributed to thin-film interference effects. This phenomenon is further corroborated by optical images within the manuscript, where a noticeable hue shift is observed across the planar sample. It is worth highlighting here that the inversion of the optical response of the nanocomposite from broadband reflectance on a transparent substrate to broadband absorbance on a reflective surface stem from the polarizonic interference that has been discussed elsewhere ^1,2^.

| 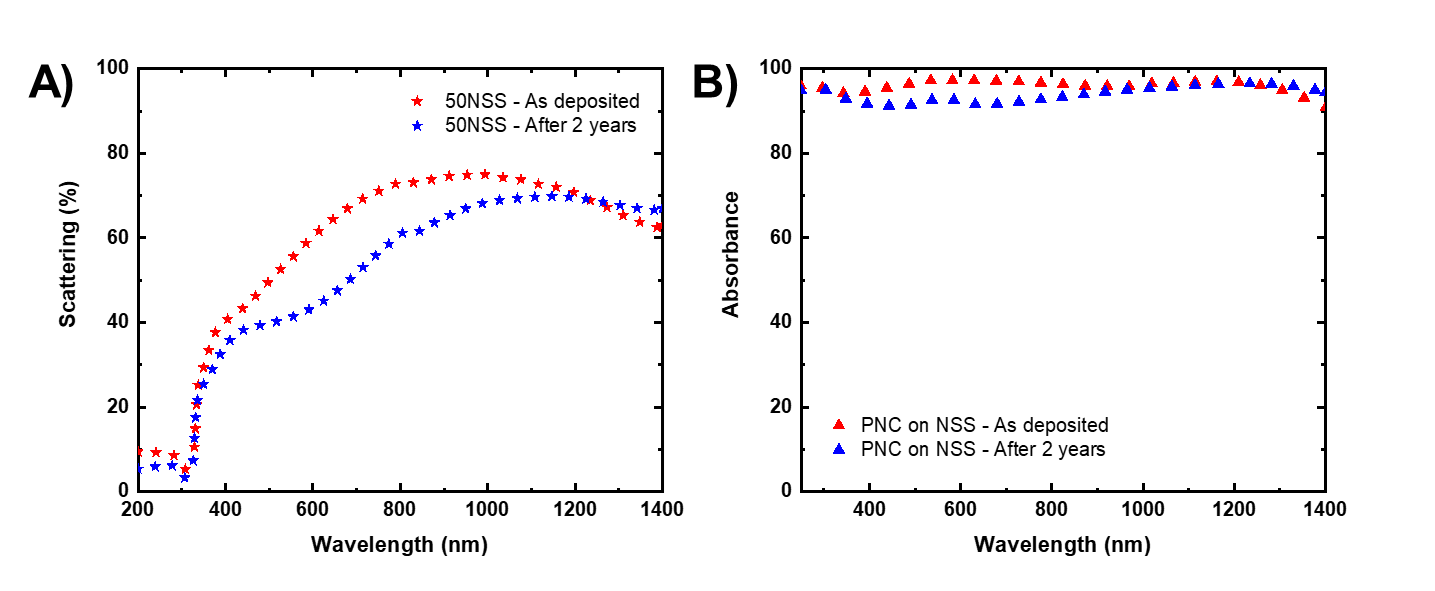 |
| --- |
| Figure S6. Stability analysis of the white and grey metasurfaces after 2 years. A) comparison of scattering spectra between the as-deposited white sample (red) and after 2 years (blue). B) comparison of the absorbance spectra of the as-deposited grey samples (red) and after 2 years (blue). |

The stability of the fabricated metasurface is crucial for practical applications, therefore the optical performance of the white sample composed of 50 nm NSS and the grey sample, composed of 70nm PNC atop 50nm PNC was measured after 2 years in storage under ambient conditions. For the white metasurface, it is evident that the Ag-based metasurface experienced a slight redshift, accompanied by approximately a 10% reduction in scattering efficiency over two years. These minor changes fall within the expected performance variations due to long-term environmental exposure. Importantly, the overall functionality and optical performance of the metasurface remain robust, ensuring effective thermal management and consistent color quality. This degree of stability is well within acceptable ranges for practical applications, underscoring the viability of our design for consumer products. Moreover, for the grey sample, the PNC layer effectively protected the NSS, resulting in the optical performance remaining nearly unchanged, with no significant alteration in its optical behavior. These results highlight the practical stability of the fabricated metasurfaces.

| 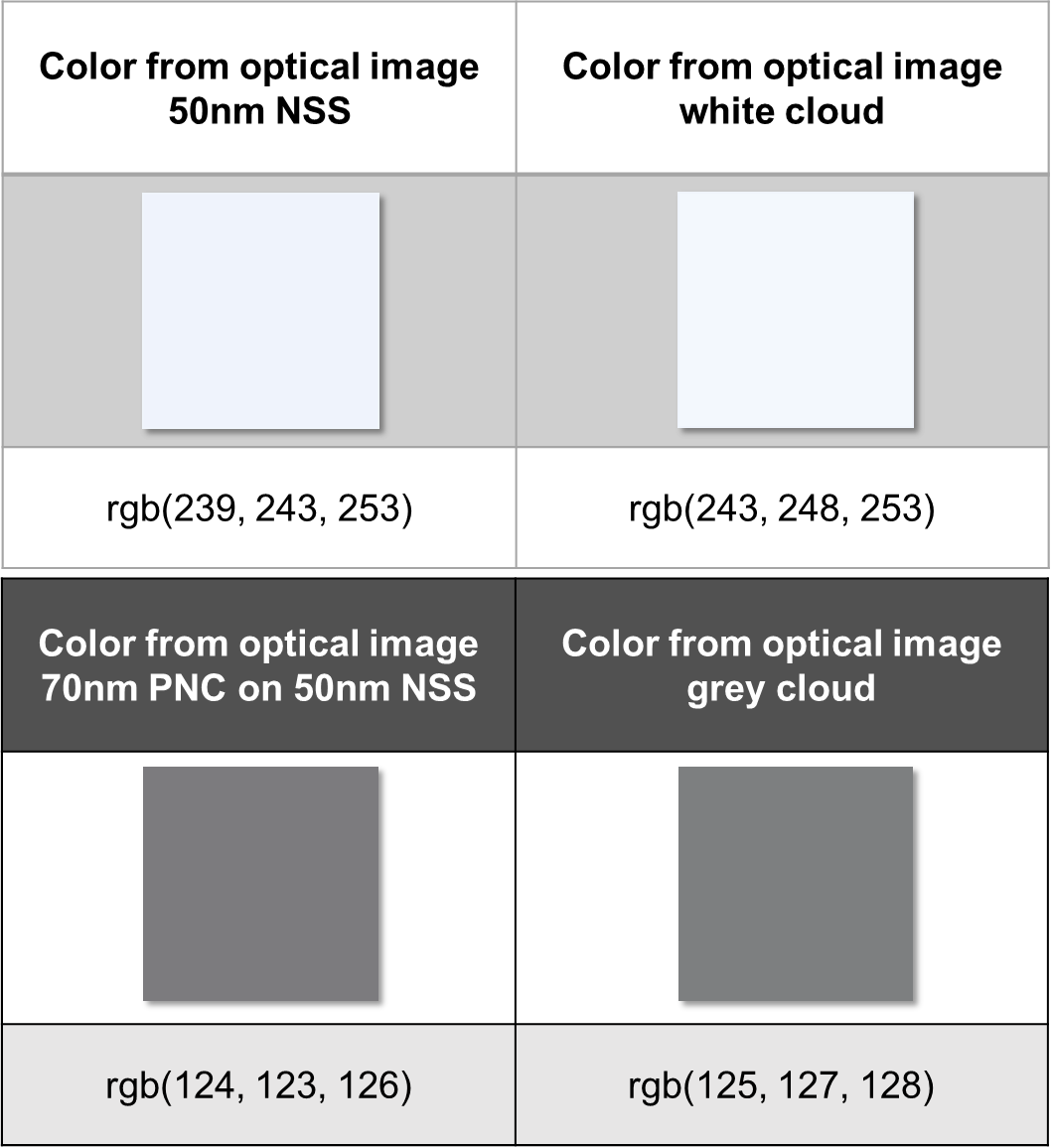 |
| --- |
| Figure S7.RGB coordinates of the fabricated samples in comparison to their surrounding environments highlighting their camouflage ability. |

**References**

1. M. Elbahri, S. Homaeigohar, M. A. Assad, Adv. Photonics Res. 2021, 2, 210000
2. M. A. Assad, M. Elbahri, Adv. Funct. Mater. 2024, 2418271
